# Impact and outcomes of hyperglycemia among post-operative children who underwent repair of congenital cardiac defects in a tertiary care setup of Pakistan

**DOI:** 10.12669/pjms.40.10.8763

**Published:** 2024-11

**Authors:** Yumna Aslam, Ayaz Ur Rehman, Tarab Zehra, Noor Ul Ain

**Affiliations:** 1 Yumna Aslam, MBBS. Department of Pediatric Medicine and Child Health, Aga Khan University Hospital, Karachi, Pakistan; 2 Ayaz Ur Rehman, MBBS. Department of Pediatric Medicine and Child Health, Aga Khan University Hospital, Karachi, Pakistan; 3 Tarab Zehra, MBBS. Department of Pediatric Medicine and Child Health, Aga Khan University Hospital, Karachi, Pakistan; 4 Noor Ul Ain, MBBS, FCPS. Department of Emergency Medicine, Aga Khan University Hospital, Karachi, Pakistan

**Keywords:** Congenital Cardiac Defects, Hyperglycemia, Pediatrics

## Abstract

**Objective:**

To determine frequency of postoperative hyperglycemia in children who underwent repair of Congenital Cardiac Defects (CCDs) in a tertiary care hospital of Pakistan. The other objective was to compare outcomes in children with versus without postoperative hyperglycemia who underwent repair of CCDs in a tertiary care hospital of Pakistan.

**Methods:**

A descriptive study was conducted at the Cardiac Intensive Care Unit (CICU), Aga Khan University Hospital (AKUH), Karachi, Pakistan from October 3, 2020, to April 2, 2021. A total of 185 patients aged one month-18 years admitted to CICU underwent elective surgical repair of CCDs meeting the eligibility criteria were included. Glucose monitoring was collected at immediate, 4^th^ and 8^th^ postoperative hours on the first day and once daily on postoperative days Two and Three to assess hyperglycemia. Data including age, weight, gender, pre-operative diagnosis, type of surgery and variables such as CICU stay, duration of hospital stay and duration of mechanical ventilation were recorded.

**Results:**

The mean age of the patients was 40.40±48.63 months. Out of 185 patients, 115 (62.2%) were male while 70 (37.8%) were female. Hyperglycemia was found to be in 182 (98.4%) patients. Out of 182 hyperglycemic patients, 53 (28.6%) had moderate hyperglycemia while 129 (69.7%) had severe hyperglycemia. Mean duration of hospital stays was 9.24±7.45, mean duration of pediatric CICU stays was 141.61±169.02 hours and mean duration of mechanical ventilation was 80.59±98.54 hours.

**Conclusion:**

Hyperglycemia is a frequent finding in children who underwent repair of CCDs, and it is not significantly associated with duration of mechanical ventilation, length of hospital and CICU stay.

## INTRODUCTION

Advances in medical sciences and postoperative care management has remarkably improved survival of children undergoing cardiac repair for Congenital Cardiac defects (CCDs).^1^ Critically ill children admitted to Pediatric Intensive Care Unit (PICU) are frequently found to be hyperglycemic.^2^-^4^ It is mainly due to endogenous stress hormone release (epinephrine, glucagon, growth hormone and cortisol), oxidative stress and inflammatory mediators.^2^ Hyperglycemia is associated with endothelial, immune, neuronal, mitochondrial dysfunction and is thought to be pro-inflammatory which may lead to end organ injury and prolonged ventilation.^5^

Study conducted in Children Hospital Boston has shown hyperglycemia contributing to increased postoperative morbidity and mortality.^6^ It was found that greater duration of time with blood glucose levels >126 mg/dl during first 72 hours was associated with prolonged length of hospitalization stay.^6^ One study conducted in Toronto revealed that hyperglycemia occurred in 90% who underwent cardiac surgery and duration, timing and intensity of hyperglycemia were associated with increased morbidity, prolonged ventilation time, longer intensive care unit and hospital stay.^7^ However, there are equivocal studies that report that hyperglycemia has no detrimental effects in infants undergoing cardiac surgery.^8^ Some studies have shown a relationship between hyperglycemia and adverse outcomes,^9^ whereas others showed that hyperglycemia was not detrimental.^10^ So, the management of hyperglycemia in the postoperative period remains controversial.^11^

In the current study, our primary aim was to determine frequency of postoperative hyperglycemia in children who underwent repair of CCDs in a tertiary care hospital of Pakistan. Our secondary aim was to determine whether there was an association between postoperative hyperglycemia length of hospital stay, duration of mechanical ventilation (MV) and length of PICU stay.

## METHODS

This prospective descriptive study was carried out over a period of six months from October 3, 2020 to April 2, 2021. A total of 185 patients aged one month-18 years admitted to the Cardiac Intensive Care Unit (CICU) underwent elective surgical repair of CCDs like unrepaired cyanotic defects including Tetralogy Of Fallot (TOF), Total Anomalous Pulmonary Venous Return (TAPVR), Tricuspid Atresia (TA) and unrepaired acyanotic defects like Atrial Septal Defects (ASD), Ventricular Septal Defects (VSD), Atrio-Ventricular Septal defects (AVSD), Coarctation of Aorta (CoA) and Patent Ductus Arteriosus (PDA) meeting the eligibility criteria were included via non-probability consecutive sampling technique. Children with active pre-operative infection at the time of presentation, renal dysfunction at the time of presentation, hepatic dysfunction at the time of presentation, preexisting diagnosis of Diabetes mellitus or Cushing Syndrome, patients receiving any type of corticosteroid (dexamethasone, prednisolone, methylprednisolone, or hydrocortisone) on the day before or on the day of surgery and/or presence of hyperglycemia on admission were excluded from the study.

### Ethical Approval:

The study was approved by Ethical Review Committee of Aga Khan Hospital, Karachi (ERC# 2020-4801-12881, dated August 26, 2020).

Data including age, weight, gender, pre-operative diagnosis, type of surgery and variables such as CICU and duration of hospital stay and duration of mechanical ventilation were recorded on a structured proforma. Any reading of blood glucose > 126 mg/dl^12^ of the first three readings taken at immediate, 4th, and 8th postoperative hours on the first day and once daily on postoperative day two and day three, measured by a calibrated ACCU-Chek Active blood glucose meter at bedside (measured by finger prick) was labeled as hyperglycemia. The severity of hyperglycemia was further stratified into moderate (126-199 mg/dl) and severe (>200 mg/dl). It was a standard of care to measure blood glucose levels after surgery every 1, 4, and 8 hourly by a calibrated ACCU-Chek Active in Joint Commission International Accreditation (JCIA) and International Organization for Standardization (ISO) 9001: 2008 certified Aga Khan University Hospital.

At the time of admission, weight of the child was measured preoperatively with light clothes and without shoes and diaper on a calibrated weighting machine by an area assigned nurse. Length was measured at the time of admission preoperatively, by an area assigned nurse for children <2 years, without shoes and headgear, with two fixing points, on a calibrated Detecto stadiometer, taking into consideration the standard protocols. Height was measured for children >2 years, by an area assigned nurse, without shoes and head gear, taking into considerations the standard protocols. Body Mass Index was calculated by using formula: Weight in kg/Height or length per meter square.

### Statistical Analysis:

The data was analyzed using SPSS version 26. For quantitative variables such as age, weight, height, body mass index (BMI), blood sugar, hospital stay, CICU stay and duration of mechanical ventilation, mean and standard deviation were reported. Frequency and percentage were calculated for categorical variables such as gender, hyperglycemia, severity of hyperglycemia, type of surgery. Outcomes were compared between hyperglycemic and non-hyperglycemic children by using independent t-test.

## RESULTS

In this study 185 patients were included to assess the postoperative hyperglycemia in children who underwent repair of CCDs and compare their outcomes in children with versus without postoperative hyperglycemia and the results were analyzed. Mean age of the patients was 40.40±48.63 days with mean weight was 10.66±8.37 kg, mean height was 81.74±25.35 cm and mean BMI was 5.911±2.50 kg/m^2^. The blood sugar at 1st, 4th and 8th hour were 209.75±99.04, 204.14±104.008 and 176.56±79.48 respectively. Besides, mean blood sugar at 2nd and 3rd day was 150.56±73.50 and 139.53±61.88 respectively.

Out of 185 patients, 115 (62.2%) were male while 70 (37.8%) were female. In distribution for type of surgery, VSD repair was noted in 75 (40.6%), TOF repair in 25 (13.5%), ASD repair 35 (18.9%), TA repair six (3.2%), Glenn repair six (3.2%), Modified Blalock-Taussig-Thomas Shunt (MBT) repair eight (4.4%), Fontan procedure three (1.6%), AVSD repair 10 (5.5%), CoA repair six (3.2%), TAPVR repair five (2.7%), Mitral Valve (MV) repair five (2.7%) while Rastelli repair was noted in one (0.5%) patient as shown in Table-I.

**Table-I:** Demographic data of n=185.

***Demographic Data***	
Age (months)	40.40 ± 3.57
Weight (kg)	10.66 ± 8.37
Height (cm)	81.74 ± 25.3
BMI (kg/m^2^)	5.9 ± 2.5
Blood Sugar (mg/dl) at 1^st^ hour	209.75 ± 99.04
Blood Sugar (mg/dl) at 4^th^ hour	204.14 ± 104
Blood Sugar (mg/dl) at 8^th^ hour	176.56 ± 79.48
Blood Sugar (mg/dl) at 2^nd^ day	150.56 ± 73.5
Blood Sugar (mg/dl) at 3^rd^ day	139.53 ± 61.8
***Gender***	
Male	115 (62.2%)
Female	70 (37.8%)
***Type of Surgery***	
VSD Repair	75 (40.6%)
TOF Repair	25 (13.5%)
ASD Repair	35 (18.9%)
TA Repair	6 (3.2%)
Glenn Shunt	6 (3.2%)
MBT Shunt	8 (4.4%)
Fontan procedure	3 (1.6%)
AVSD Repair	10 (5.5%)
CoA Repair	6 (3.2%)
TAPVR Repair	5 (2.7%)
MV Repair	5 (2.7%)
Rastelli	1 (0.5%)

Hyperglycemia was found to be in 182 (98.4%) patients. Out of 182 hyperglycemic patients, 53 (28.6%) had moderate hyperglycemia while 129 (69.7%) had severe hyperglycemia. Mean duration of hospital stays was 9.24±7.45, mean duration of pediatric CICU stays was 141.61±169.02 hours and mean duration of mechanical ventilation was 80.59±98.54 hours, (Table-II).

**Table-II:** Frequency of postoperative hyperglycemia and outcomes in children who underwent repair of congenital cardiac defects.

Postoperative Hyperglycemia And Outcomes	Statistics
***Hyperglycemia***	
Yes	182 (98.4%)
No	3 (1.6%)
***Severity of Hyperglycemia (n = 182)***	
Moderate	53 (2.86%)
Severe	129 (69.7%)
Hospital Stay (days)	9.24 ± 7.45
CICU Stay (hours)	141.61 ± 169.05
Mechanical Ventilation (hours)	80.59 ± 98.54

In comparison of postoperative outcomes with or with hyperglycemia, mean±SD of length of hospital stays was noted as 9.32±7.48 & 4.67±1.52 having a non-significant P-value i.e., (0.285), length of CICU stays 143.51±169.75 & 26.50±18.70 while duration of mechanical ventilation was noted as 81.74±98.95 & 11.33±4.93 among hyperglycemic & non-hyperglycemic patients having a non-significant p-value i.e., (0.235) & (0.221) respectively, (Table-III).

**Table-III:** Comparison of postoperative outcomes with versus without hyperglycemia n=185.

Outcomes		Hyperglycemia		P-value

		Yes (n=182)	No (n=3)	
Length of Hospital Stays In Days	Mean	9.32	4.670	0.285
	±SD	±7.489	±1.528
Length of CICU Stays In Hours	Mean	143.51	26.50	0.235
	±SD	±169.75	±18.70
Duration of Mechanical Ventilation In Hours	Mean	81.74	11.33	0.221
	±SD	±98.95	±4.93
ANOVA	0.0001			

Applied Independent t-test & ANOVA test.

## DISCUSSION

The findings of this study reflect that hyperglycemia is a very common complication in children admitted to PICU. Most of the children were severely hyperglycemic. However, hyperglycemia was not associated with adverse outcomes such as increased duration of mechanical ventilation, and prolonged length of hospital and CICU stay. Although in adults higher blood glucose levels in the intraoperative and postoperative period of cardiac surgery are associated with higher morbidity and mortality,^10^,^11^ little is known about that relation in the pediatric population. A large number of possible confounding factors, such as prematurity of the physiological systems, greater availability of surgical procedures, and less uniformity in preoperative clinical conditions, make conclusive results difficult to obtain.

In this study, we confirmed that hyperglycemia is a common event after pediatric cardiac surgery. Although different cut-off values were defined for hyperglycemia in different studies, the incidence of hyperglycemia has been reported to range from 43 to 98%^2^,^8^. Similar to those studies, 98.4% of our patients were hyperglycemic and 69.7% of these patients had severe hyperglycemia. In our study, mean age was 40.40±48.63 months. Alaei F, et al reported age in months as 52.74±46.42 10. Another study found age to be 10 months.^13^ In present study, male population was dominant as 115 (62.2%) were male while 70 (37.8%) were female. Ödek Ç et al. found to have 49% males.^14^ The difference in findings may be as above quoted studies were conducted in developed countries.

In current study, mean duration of hospital stays was 9.24±7.45 days, mean duration of pediatric CICU stays was 141.61±169.02 hours and mean duration of mechanical ventilation was 80.59±98.54 hours. Alaei F et al. documented the length of stay at hospital to be 17.01±11.21 days, duration of PICU as 103.12±185.26 hours and the duration of mechanical ventilation as 50.28±177.79 hours.^13^ Upon comparison of length of hospital stay, duration of PICU and duration of mechanical ventilation between hyperglycemic and non-hyperglycemic group, no significant difference was observed in our study whereas, Yates and co-workers has stated in its study that duration of hyperglycemia was also significantly associated with increased intensive care (*p* <0.001) and hospital (*p* < .001) stay and longer ventilator use (*p* <0.001).^15^

In a study published in 2014, S. Hussain et al. reported that 87 (1.5%) neonates were found to have CCDs with an incidence of 15/1000 in a tertiary care setup of Pakistan.^16^ Prevalence of CCDs in rural Pakistan is quite high as compared to urban facility based data and it leads to high infant mortality as indicated by sharp fall in prevalence after one year.^17^

Hyperglycemia is a regular phenomenon in critically ill children after surgical repair or palliation of CCDs. Some recent studies have shown an association of hyperglycemia with increased postoperative morbidity and mortality in these children.^18^

### Limitations:

This study has several limitations which includes small sample size, study period, single centered study and use of non-probability sampling technique.

## CONCLUSION

Hyperglycemia is a frequent finding in children who underwent repair of CCDs, and it is not significantly associated with duration of mechanical ventilation, length of hospital and CICU stay. Timely diagnosis and correction of hyperglycemia can lead to decrease disease morbidity and duration of hospitalization. More studies with larger sample size and better study design need be conducted in order to draw a concrete conclusion.

### Authors Contribution:

**YA and AUR:** Concept, design, interpretation and analysis of data, and writing and final revision of manuscript. Responsible for data accuracy and accountable for the integrity of the manuscript. **TZ:** Data collection, data entry, and final revision of manuscript. **NA:** Data analysis and interpretation, manuscript writing. All authors have read and approved final version of manuscript.
